# Suppression of Heregulin-**β**1/HER2-Modulated Invasive and Aggressive Phenotype of Breast Carcinoma by Pterostilbene via Inhibition of Matrix Metalloproteinase-9, p38 Kinase Cascade and Akt Activation

**DOI:** 10.1093/ecam/nep093

**Published:** 2011-02-14

**Authors:** Min-Hsiung Pan, Ying-Ting Lin, Chih-Li Lin, Chi-Shiang Wei, Chi-Tang Ho, Wei-Jen Chen

**Affiliations:** ^1^Department of Seafood Science, National Kaohsiung Marine University, Nan-Tzu, Kaohsiung, Taiwan; ^2^Department of Biomedical Sciences, Chung Shan Medical University, No. 110, Section 1, Chien-Kuo N. Road, Taichung 402, Taiwan; ^3^Institute of Medicine, Chung Shan Medical University, Taichung 402, Taiwan; ^4^Department of Food Science, Cook College, Rutgers University, New Brunswick, NJ, USA

## Abstract

Invasive breast cancer is the major cause of death among females and its incidence is closely linked to HER2 (human epidermal growth factor receptor 2) overexpression. Pterostilbene, a natural analog of resveratrol, exerts its cancer chemopreventive activity similar to resveratrol by inhibiting cancer cell proliferation and inducing apoptosis. However, the anti-invasive effect of pterostilbene on HER2-bearing breast cancer has not been evaluated. Here, we used heregulin-**β**1 (HRG-**β**1), a ligand for HER3, to transactivate HER2 signaling. We found that pterostilbene was able to suppress HRG-**β**1-mediated cell invasion, motility and cell transformation of MCF-7 human breast carcinoma through down-regulation of matrix metalloproteinase-9 (MMP-9) activity and growth inhibition. In parallel, pterostilbene also inhibited protein and mRNA expression of MMP-9 driven by HRG-**β**1, suggesting that pterostilbene decreased HRG-**β**1-mediated MMP-9 induction via transcriptional regulation. Examining the signaling pathways responsible for HRG-**β**1-associated MMP-9 induction and growth inhibition, we observed that pterostilbene, as well as SB203580 (p38 kinase inhibitor), can abolish the phosphorylation of p38 mitogen-activated protein kinase (p38 kinase), a downstream HRG-**β**1-responsive kinase responsible for MMP-9 induction. In addition, HRG-**β**1-driven Akt phosphorylation required for cell proliferation was also suppressed by pterostilbene. Taken together, our present results suggest that pterostilbene may serve as a chemopreventive agent to inhibit HRG-**β**1/HER2-mediated aggressive and invasive phenotype of breast carcinoma through down-regulation of MMP-9, p38 kinase and Akt activation.

## 1. Introduction

Invasive breast cancer is the most frequent cause of death among women in the industrialized world [1] and its incidence tightly correlates with genetic abnormalities such as HER2 (human epidermal growth factor receptor 2). Numerous studies have shown that HER2-positive breast cancer is highly proliferative and invasive with metastatic potential [2–5]. A literature reviewing 81 studies and 27 161 patients with respect to HER2 reveals that amplification or overexpression of HER2 is closely linked to axillary-lymph-node metastases with poor outcome, which protrudes the prognostic value of HER2 in evaluating the development of invasive breast carcinoma [6].

Molecular mechanisms by which HER2 deregulation promotes cell invasion and metastasis of breast carcinoma are less known yet. However, several investigations have provided information on the biology of breast cancer dissemination in response to HER2 hyperactivation. Reduction of HER2 activity or expression leads to down-regulation of Akt and ERK1/2 signaling and, consequently, represses the expression of angiopoietin-2, an initial factor required for tumor angiogenesis, indicating that HER2 mediates tumor angiogenesis and metastasis by up-regulation of angiopoietin-2 expression [7]. In HER2-overexpressing cells, transforming growth factor-*β* activates phosphotidylinositol 3-kinase (PI3K), and then recruits and stabilizes actin/actinin/HER2 complex at cell protrusions, which sustains prolonged Rac1 activation, and enhances cell motility and invasiveness [8]. HER2 activity may assist breast cancer cells in homing to specific metastatic organs via enhancement of chemokine receptor CXCR4 expression or stability [9]. Using phosphor-specific antibodies against c-Src and focal adhesion kinase (FAK), demonstrates that HER2 signaling selectively increases Tyr phosphorylation of c-Src at Tyr-215 and Tyr phosphorylation of FAK at Tyr-861, resulting in the spread of breast cancer cells by HER2 [10]. Furthermore, co-culturing MCF-7 breast cancer cells overexpressing HER2 with endothelial cell monolayers displays that the increased metastatic potential of breast carcinoma may stem from HER2 induction of endothelial cell retraction, a process disrupting endothelial integrity and preceding endothelial cell transmigration in several tumor metastatic models [11], and the plausible mechanism involves both down-regulation of vascular endothelial cadherin and dissociation of catenins by HER2 [12]. Totally, tests in both cell-based and animal models confirm a critical role of HER2 hyperactivation in the development of breast carcinoma cell invasion and metastasis, consistent with the observations from clinic studies.

Other possibility that HER2 amplification or overexpression contributes to breast cancer metastasis is up-regulation of matrix metalloproteinases (MMPs), a family of zinc and calcium-dependent endoproteinases responsible for the degradation of extracellular matrix (ECM). A causal link between MMPs and HER2 has been deciphered by clinicopathological evidence. Immunohistochemical and statistical analysis found that MMP-2 and MMP-9 expressions are positively related to HER2 overexpression in the stromal cells of breast carcinoma [13], implicating that MMP-2 or MMP-9 may be a responsive gene of HER2 signaling in breast cancer metastasis. Several cell-based studies can provide the molecular basis of the axis of HER2 signaling to MMP-9 expression in breast carcinoma. Introduction of HER2 into human MCF-10A breast cancer cells, a non-tumorigenic epithelial cell line, induced MMP-9 up-regulation via p38 mitogen-activated protein kinase (p38 kinase) and Akt signaling pathways that further enhanced the invasive and migratory capacities of MCF-10A cells [14]. Heregulin-*β*1 (HRG-*β*1), a natural epidermal growth factor (EGF)-like ligand for HER3 and HER4 [15] and emits its signaling by transactivating HER2 [16], is capable of elevating MMP-9 expression and secretion via activation of several major HER2-mediated downstream signaling pathways, including mitogen-activated protein kinase (MAPK) cascade, p38 kinase cascade and phospholipase C*γ*-protein kinase C (PLC*γ*-PKC) pathways [17]. On basis of these studies, it is suggested that MMP-9 may be a valuable target of therapeutic intervention in breast cancer patients whose HER2 are overexpressed.

**Figure 1:**
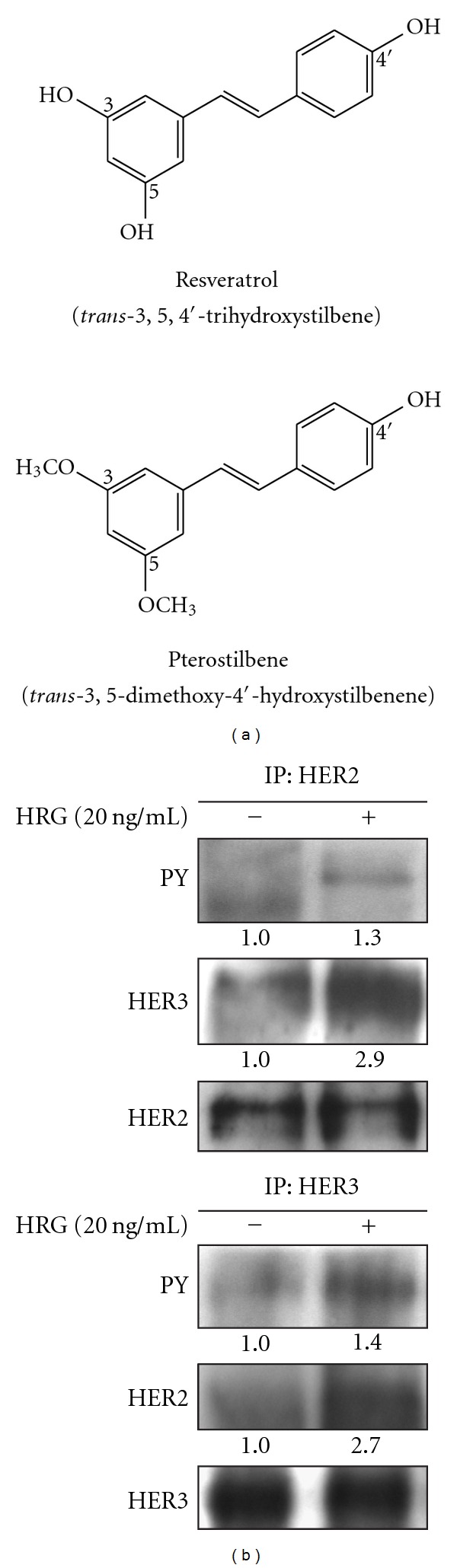
(a) Chemical structures of resveratrol and pterostilbene. (b) Transactivation of HER2 by heterodimerizing HER3 in response to HRG-*β*1. HRG-*β*1-stimulated cell lysates were immunoprecipitated with HER2 or HER3 antibodies, and then immunoblotted for antibodies against phosphotyrosine (PY), HER3 or HER2. HRG: heregulin-*β*1; IP: immunoprecipitation.

Pterostilbene (*trans*-3,5-dimethoxy-4′-hydroxystilbene) (Figure 1(a)), a dimethyl ether analog of resveratrol, belongs to the naturally occurring stilbene phytoalexin and can be isolated from some types of berries [18–20]. Pterostilbene has been suggested to possess anti-neoplastic activity as effective as resveratrol due to their closely structural similarity [21–24]. Pterostilbene also acts as an iNOS inhibitor against aberrant crypt foci formation in the azoxymethane-induced colon carcinogenesis in rats [25] and down-regulates lipopolysaccharide (LPS)-induced iNOS and COX-2 expression of murine macrophage [26].

The anti-cancer activities of pterostilbene have, over the years, attracted the attention of many researchers; however, the inhibitory effect of pterostilbene on HER2-mediated breast cancer cell invasion has not yet been investigated. To this end, we used HRG-*β*1 to transactivate HER2 signaling and investigated whether pterostilbene inhibits HER2-mediated invasion, metastasis and MMP-9 expression of breast carcinoma. We also examined the effects of pterostilbene on several major HER2-mediated downstream signaling pathways responsible for MMP-9 expression. Then, we tried to define the cancer-preventive action of pterostilbene in HER2-positive breast carcinoma.

## 2. Methods

### 2.1. Materials

Recombinant human heregulin-*β*1 was purchased from R&D Systems (Minneapolis, MN, USA). Pterostilbene, p38 kinase inhibitor SB203580, MEK inhibitor PD98059 and PI3K inhibitor LY294002 were obtained from Sigma (St Louis, MO, USA). RT–PCR reagents were from Promega (Madison, WI, USA). The antibodies against *β*-actin, HER3 and HER2 were obtained from Santa Cruz Biotechnology (Santa Cruz, CA, USA). Antibodies against phospho-p38 (Thr180/Tyr182), p38, phospho-ERK1/2 (Thr42/Tyr44), ERK1/2, phospho-Akt (Ser473) and Akt were from Cell Signaling Technology (Beverly, MA, USA). The anti-phosphotyrosine (PY) antibody (4G10) was available from Upstate Biotechnology (Charlottesville, VA, USA).

### 2.2. Cell Culture

Monolayer cultures of MCF-7, a HER2-positive human breast carcinoma, were grown in Dulbecco's minimal essential medium (DMEM) supplemented with 10% fetal calf serum (Gibco BRL, Grand Island, NY, USA), 100 units/ml of penicillin and 100 *μ*g/ml of streptomycin, and kept at 37°C in a humidified atmosphere of 5% CO_2_ in air.

### 2.3. Immunoprecipitation

One hundred microliter cell lysate (containing 500*μ*g total cellular proteins) was first precleared by being incubated with protein A-agarose (10 *μ*l, 50% slurry, Santa Cruz Biotechnology) for 15 min. The clarified supernatants were collected by microfugation, and then incubated with primary antibody for 2 h at 4°C. The reaction mixtures were added with 20 *μ*l of protein A-agarose to absorb the immunocomplexes at 4°C overnight. Immunoprecipitated proteins were subjected to 8% SDS–PAGE, and then transferred onto a PVDF membrane (Millipore). The native- or phospho-form proteins were visualized by immunoblotting.

### 2.4. Western Blotting (Immunoblotting)

Western blotting was performed as previously described [27]. Briefly, total protein extracts were prepared in a lysis buffer (50 mM Tris–HCl, pH 8.0, 5 mM EDTA, 150 mM NaCl, 0.5% NP-40, 0.5 mM phenylmethanesulfonyl fluoride and 0.5 mM dithiothreitol) for 30 min at 4°C. Equal amounts of total cellular proteins (50 *μ*g) were resolved by SDS–PAGE, transferred onto polyvinylidene difluoride (PVDF) membranes (Immobilon P, Millipore, Bedford, MA, USA) and then probed with primary antibody, followed by secondary antibody conjugated with horseradish peroxidase. The immunocomplexes were visualized with enhanced chemiluminescence kits (Amersham, UK).

### 2.5. In Vitro Invasion Assay

The migratory ability of the cells was assayed in Transwell upper and lower chambers (Costar, Cambridge, MT, USA) separated by a polycarbonate membrane (8-mm pores, 6.5 mm diameter). Before invasion assays, Matrigel matrix (BD Biosciences, Bedford, MA, USA) was diluted 80 times with serum-free cold DMEM. Then the polycarbonate filter was coated with 100 *μ*l of diluted Matrigel matrix and allowed to dry overnight. For invasion assays, 2.5 × 10^4^ serum-starved MCF-7 cells seeded in the upper well of each chamber were treated with a variety of concentrations of pterostilbene accompanied with HRG-*β*1 (20 ng/ml) stimulation, and NIH3T3 fibroblast-conditioned media was placed in the lower compartment of the chemotaxis chamber as a source of chemoattractants. The invasion was allowed to proceed for 72 h, followed by fixation with 10% formaldehyde for 10 min and staining with 0.25% Coomassie Brilliant Blue G. The number of cells invading the lower side of the filter was counted under microscopy in 10 random selective fields.

### 2.6. Wound-Healing Assay

A cell culture wound-healing assay was performed following well-established methods [28]. To study the effects of pterostilbene on HRG-*β*1-induced cell migration, serum-starved MCF-7 cells were grown to confluence and a linear wound was created in the confluent monolayer using a 200 *μ*l micropipette tip. The cells were then washed with PBS to eliminate detached cells and diluted in serum-free DMEM. Then, various concentrations of pterostilbene were added for 30 min before HRG-*β*1 (20 ng/ml) treatment. After 24 h of incubation, the wound edge movement was monitored with a microscope.

### 2.7. Anchorage-Independent Transformation Assay

Anchorage-independent transformation assay (soft agar assay) was performed as previously described [29]. Briefly, single-cell suspensions of MCF-7 cells were stimulated with 20 ng/ml of HRG-*β*1 in the presence of different concentrations of pterostilbene, and then mixed with agarose in a final concentration of 0.35%. Aliquots of 1.5 ml containing 10^4^ cells with 10% FCS were plated in triplicate in 6-cm culture dishes over a base layer of 0.7% agarose and allowed to grow. Colonies of >60 mm was counted after 14 days of incubation.

### 2.8. Cell Viability Assay

MCF-7 cells were seeded at a density of 5 × 10^3^ cells/ml into 96-well plates and grown overnight. Then the cells were transferred into serum-free medium and incubated for an additional 16 h. After serum starvation, the cells were treated with the indicated concentrations of pterostilbene for 30 min prior to stimulation with 20 ng/ml of HRG-*β*1. After 24 h of incubation, the proliferating cell numbers were determined by the MTT (3-(4,5-dimethylthiazol-2-yl)-2,5-diphenyltetrazolium bromide) assay. Briefly, 20 *μ*l of MTT solution (5 mg/ml, Sigma) was added to each well and incubated for 4 h at 37°C. Then the supernatant was aspirated, and the MTT-formazan crystals formed by metabolically viable cells were dissolved in 200 *μ*l of dimethyl sulfoxide (DMSO). Finally, the absorbance was monitored by a microplate reader at a wavelength of 570 nm.

### 2.9. Gelatin Zymographic Assay

Cultures deprived of serum for 24 h were treated with the increasing doses of pterostilbene (0, 5, 10, 20 or 30 *μ*M) for 30 min prior to stimulation with HRG-*β*1 (20 ng/ml). After 24 h, conditioned media were concentrated 50-fold using Centricon spin columns (Amicon, Beverly, MA, USA), mixed with Laemmli's sample buffer in the absence of *β*-mercaptoethanol, and then subjected to 10% SDS–PAGE with gels containing 0.1% gelatin (Sigma). After electrophoresis, the gel was washed twice in a solution containing 2.5% Triton X-100 to remove SDS and subsequently incubated in 50 mM Tris–HCl (pH 7.5) buffer containing 10 mM calcium chloride and 1 mM zinc chloride with shaking for 24 h at 37°C. The gelatinolytic activity were visualized after staining the gel with 0.25% Coomassie blue in 10% acetic acid and 45% methanol and then destaining in 10% acetic acid and 5% methanol.

### 2.10. Reverse Transcription Polymerase Chain Reaction

Total RNA was isolated using TRIzol reagent (Invitrogen) as recommended by the manufacturer's instructions. Total RNA (5 *μ*g) was reverse-transcribed into cDNA, using Moloney murine leukemia virus (M-MLV) reverse transcriptase and oligo (dT) 18 primer by incubating the reaction mixture (25 *μ*l) at 40°C for 90 min. Amplification of cDNA was performed by polymerase chain reaction (PCR) in a final volume of 50 *μ*l containing 2 *μ*l of reverse transcription (RT) product, dNTPs (each at 200 *μ*M), 1× reaction buffer, a 1-*μ*M concentration of each primer (MMP-9, forward 5′-GGAGCCGCTCTCCAAGAAGCTT-3′, reverse 5′-CTCCTCCCTTTCCTCCAGAACAGAA-3′; and GAPDH, forward 5′-TGAAGGTCGGTGTGAACGGATTTGGC-3′, reverse 5′-CATGTAGGCCATGAGGTCCACCAC-3′) and 50 units/ml Pro *Taq* DNA polymerase. After an initial denaturation for 5 min at 95°C, 30 cycles of amplification (95°C for 30 s, 58°C for 1 min and 72°C for 1 min) were performed, followed by 72°C for 10 min. A 5-*μ*l sample of each PCR product was electrophoresed on a 2% agarose gel and visualized by ethidium bromide staining.

### 2.11. Statistical Analysis

Mean values between the groups were compared using the Student's unpaired two-tailed *t*-test. All statistical tests were two-sided, and differences were considered significant when *P* < .05.

## 3. Results

### 3.1. Facilitation of HER2–HER3 Heterodimer Formation in Response to HRG-*β*1 in MCF-7 Cells

A ligand for HER2 has not yet been found, but it can be transactivated by forming heterodimer with HER3 in response to HRG-*β*1 stimulation. To understand whether HRG-*β*1 can induce HER2 activation via heterodimerization with HER3 in human breast cancer cell line MCF-7, we analyzed the status of tyrosine phosphorylation of HER2 and HER3, a characteristic for RTK activation, in response to HRG-*β*1 in MCF-7 cells. HER2 or HER3 protein from HRG-*β*1-stimulated cell lysates was purified by immunoprecipitation, and then phosphorylated proteins were visualized by immunoblotting with anti-phosphotyrosine (PY) antibody (4G10 monoclonal antibody). As shown in Figure 1(b), either HER2 or HER3 tyrosine phosphorylation was markedly increased in HRG-*β*1-treated cells compared with that in HRG-*β*1-untreated control. Co-immunoprecipitation assay also showed that HRG-*β*1 promoted HER2–HER3 heterodimer formation in MCF-7 cells (Figure 1(b)). These results indicated that we could use HRG-*β*1-stimulated MCF-7 cells to mimic cellular physiology of HER2-overexpressing breast cancer cells.

### 3.2. Abrogation of HRG-*β*1-Mediated Cell Invasion by Pterostilbene in a Transwell Assay

**Figure 2:**
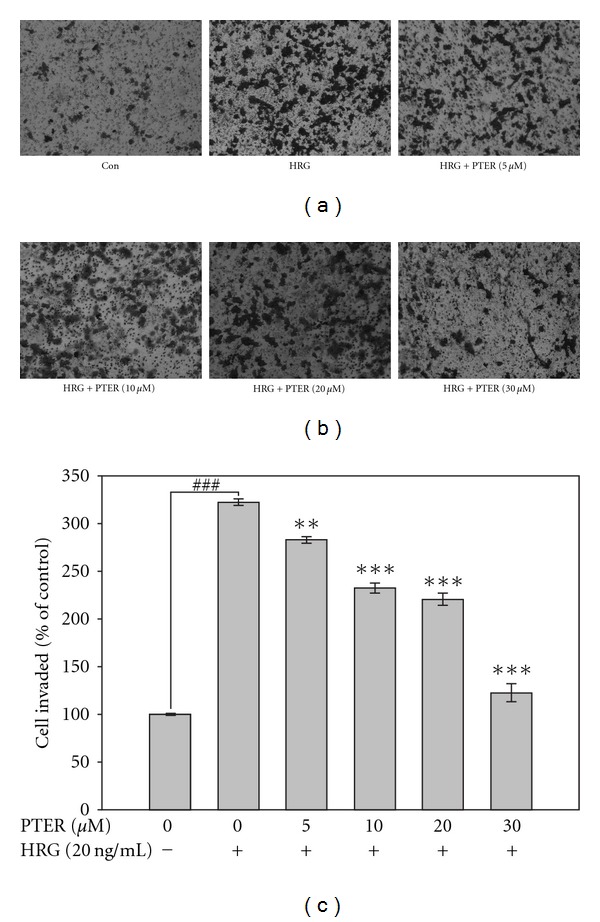
Effect of pterostilbene on HRG-*β*1-mediated human breast cancer cell invasion. Invasion of tumor cells was analyzed in Transwell Boyden chambers with a polycarbonate filter of 8-*μ*m pore size. Serum-starved MCF-7 cells were seeded in Transwell upper chamber coated with a 1 : 80 diluted Matrigel. After 12 h attachment, cells plated in upper chamber were treated with HRG-*β*1 (20 ng/ml) in the presence of the indicated concentrations of pterostilbene and allowed to invade for 72 h. Invading cells on the lower surface of filter were stained and quantified with microscope. For quantification, the number of invasive cells were counted in 10 random selective fields per well and each bar represents mean ± SE of three independent experiments. **P < .01 and ***P < .001 compared with HRG-*β*1-stimulated group; ^###^ *P* < .001 compared with serum-deprived control. PTER represents pterostilbene.

HRG-*β*1 was reported to involve in the regulation of tumorigenesis and metastasis of breast cancer [30–32]. To assess the effect of pterostilbene on HRG-*β*1-modulated invasion, HRG-*β*1-stimualted MCF-7 cells were allowed to invade through reconstituted basement membranes (Matrigel) by Transwell assay. Representative micrographs of Transwell filters are shown in Figure 2. HRG-*β*1 dramatically promoted the invasion activity of MCF-7 cells compared with that of the control, while the addition of pterostilbene to MCF-7 cells led to a dose-dependent inhibition on HRG-*β*1-mediated cell invasion. This finding revealed that pterostilbene was able to suppress HRG-*β*1-associated invasion of breast cancer cells.

### 3.3. Restriction on In Vitro Migration of HRG-*β*1-Stimulated Cells by Pterostilbene

**Figure 3:**
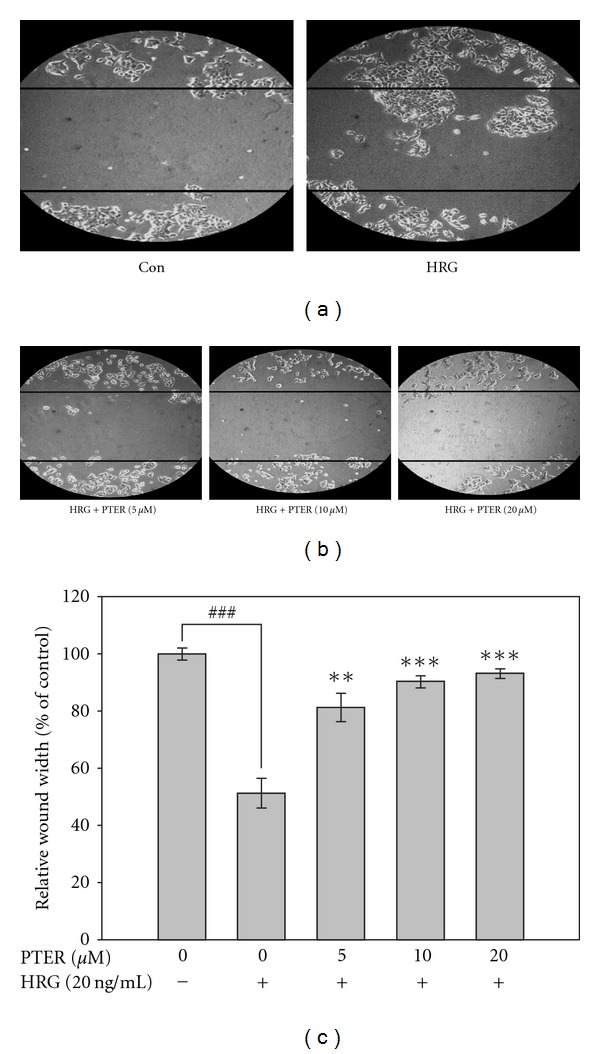
Effect of pterostilbene on HRG-*β*1-modulated wound healing. MCF-7 cells were seeded on 6-well plates, starved, and grown to confluence. A scrape wound was generated in the cell layer, and the culture was treated with pterostilbene at a concentration of 0, 5, 10 or 20 *μ*M for 30 min prior to HRG-*β*1 (20 ng/ml) stimulation. Then the cells on the edge of the wound were allowed 24 h to migrate across the wound. All of the results were monitored by microscopy. Relative distance of the wound width was measured and divided by the initial half-width of the wound. Results are expressed as the mean ± SE of three independent experiments. *P < .05, **P < .01 and ***P < .001 versus HRG-*β*1-stimulated control; ^###^ *P* < .001 compared with serum-starved control.

Next, to evaluate the effect of pterostilbene on HRG-*β*1-induced cell motility, the cell culture wound-healing assay, an established method to study directional cell migration *in vitro* [33], was employed. Incubation of serum-starved MCF-7 cells with HRG-*β*1 produced a marked cell migration in the wound area at 24 h after wounding; whereas wounds treated with pterostilbene showed dose-related delays in wound healing under the same conditions (Figure 3). The result indicated that pterostilbene inhibited HRG-*β*1-induced motility of breast cancer cells *in vitro.*

### 3.4. Retardation of HRG-*β*1-Mediated Cell Transformation by Pterostilbene

**Figure 4:**
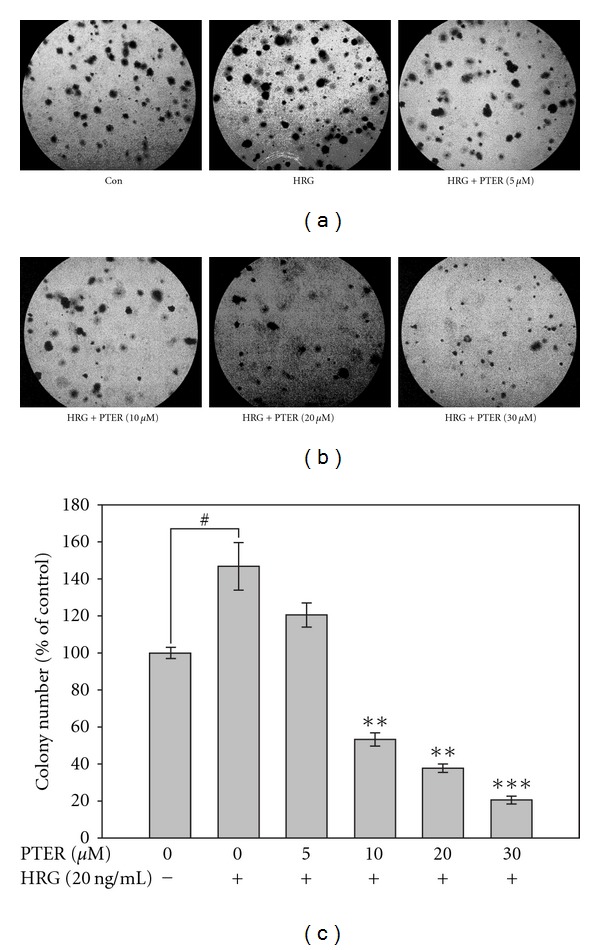
Effects of pterostilbene on colony formation of HRG-*β*1-stimulated MCF-7 cells. Serum-starved MCF-7 cells were treated with different concentrations of pterostilbene, together with stimulation by HRG-*β*1 (20 ng/ml), in 0.35% agarose containing 1% FCS over 0.7% agarose containing 1% FCS. Cell colonies were counted by light microscopy after a 14-day incubation at 37°C in 5% CO_2_. For quantification, colonies greater than 60 mm were scored. Statistical analysis was analyzed by *t-*test. **P < .01 and ***P < .001 compared with HRG-*β*1-stimulated group; ^#^ *P* < .05 compared with serum-starved control.

Anchorage-independent growth is positively linked to metastatic potential [34]. To investigate the inhibitory effects of pterostilbene on HRG-*β*1-induced tumorigenicity *in vitro*, HRG-*β*1-stimulated MCF-7 cells were seeded on a soft agar, and the transformed colonies greater than 60 *μ*m were counted. After 2 weeks of incubation, HRG-*β*1 increased about 50% of the transformed colonies compared with that of the unstimulated control, and this capacity of soft agar colony formation by HRG-*β*1 was markedly inhibited by pterostilbene with a lower number of colonies formed and a reduced colony size in a concentration-responsive manner (Figure 4). Notably, the number of colonies formed at the high doses of pterostilbene (10–30 *μ*M) was much lower than that of the unstimulated control. These results suggested that pterostilbene strongly inhibited anchorage-independent growth in HRG-*β*1-stimulated MCF-7 cells and this effect might, in part, be due to the cytotoxicity of pterostilbene.

### 3.5. Viability of HRG-*β*1-Stimulated MCF-7 Cells in the Presence of Pterostilbene

**Figure 5:**
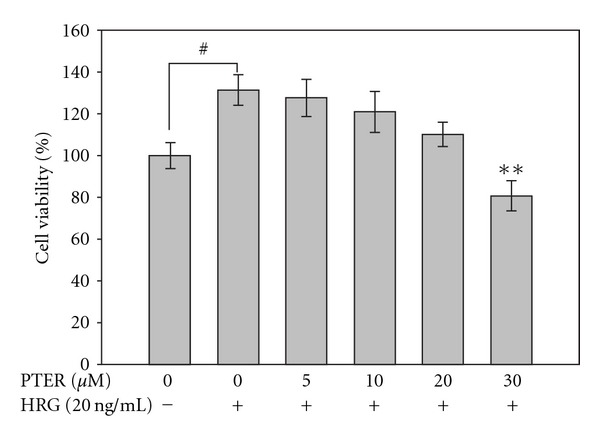
Effect of pterostilbene on HRG-*β*1-stimulated MCF-7 cell viability. Serum-starved MCF-7 cells were pretreated with 0, 5, 10, 20 or 30 *μ*M of pterostilbene for 30 min before 20 ng/ml of HRG-*β*1 was added into the culture medium. After 24 h of incubation, the cell viability was determined by the MTT assay as described in Section 2, and the results are shown as the cell number relative to that of the unstimulated cells. Each experiment was independently performed three times and expressed as the mean ± SE. **P < .01 compared with the HRG-*β*1-treated control; ^#^ *P* < .05 compared with serum-starved control.

The anti-tumorigenic effect of pterostilbene on soft agar assay led us to determine whether this inhibition by pterostilbene resulted from its cytotoxicity. Thus, the anti-proliferating activity of pterostilbene on HRG-*β*1-stimulated MCF-7 cells was examined by means of MTT method with the indicated concentrations of pterostilbene as shown in Figure 5. The result showed that pterostilbene slightly inhibited HRG-*β*1-stimulated cell proliferation, but the inhibitory efficacy of pterostilbene on cell growth was obviously lower than that on invasion and motility (Figures 2 and 3(a), resp.), indicating that growth inhibition is unsufficient to explain the down-regulation of HRG-*β*1-induced metastatic spread by pterostilbene.

### 3.6. Suppressive Effects of Pterostilbene on the Activity and Expression of MMP-9 Induced by HRG-*β*1

**Figure 6:**
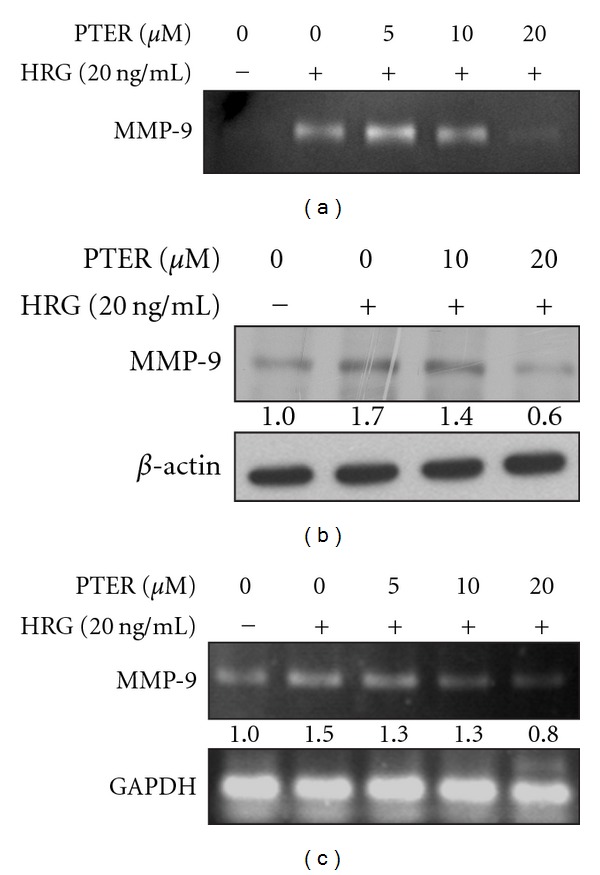
Effects of pterostilbene on HRG-*β*1-mediated MMP-9 activity and expression in MCF-7 breast cancer cells. (a) Inhibition of HRG-*β*1-mediated MMP-9 gelatinolytic activity by pterostilbene. Serum-starved MCF-7 cells were pretreated with 0, 5, 10, 20 or 30 *μ*M of pterostilbene for 30 min followed by exposure to 20 ng/ml of HRG-*β*1. After 24 h of incubation, the conditioned medium was collected and subjected to zymography. (b) Inhibition of HRG-*β*1-mediated MMP-9 protein expression by pterostilbene. Cells were pretreated with various concentrations of pterostilbene for 30 min in serum-free media and then stimulated by 20 ng/ml of HRG-*β*1 for 24 h. At the end of incubation, total proteins in cellular extracts were collected and assayed by western blotting using an antibody against MMP-9. *β*-Actin was an internal control for equivalent protein loading. (c) Dose-related inhibition of HRG-*β*1-induced increase in MMP-9 protein level by pterostilbene. Serum-starved cells were stimulated with HRG-*β*1 (20 ng/ml) for 24 h after pretreatment of pterostilbene at the indicated concentrations. Then total RNA was isolated, and RNA expression was analyzed by RT–PCR as described in Section 2. Glyceraldehyde-3-phosphate dehydrogenase (GAPDH) cDNA was used as an internal control.

MMP-9 plays important role in the facilitation of tumor invasion and metastasis, and recent evidence has shown that HRG-*β*1 can stimulate MMP-9 secretion and activation via a transcriptional regulation in human breast cancer cells [17]. Thus, to understand the anti-invasive and anti-metastatic mechanisms of pterostilbene, the possibility of pterostilbene affecting the activity and expression of MMP-9 in HRG-*β*1-activated MCF-7 cells was assessed by zymographic assay and western blot analysis, respectively. The result from zymogram gels indicated that pterostilbene down-regulated HRG-*β*1-modulated MMP-9 activation in a dose-related manner (Figure 6(a)). Subsequently, as shown by western blotting, pterostilbene blocked the expression of pro-MMP-9 protein by HRG-*β*1 in MCF-7 cells (Figure 6(b)), and the pattern of protein levels of pro-MMP-9 correlated well with that of MMP-9 gelatinolytic activity shown in zymographic assay (Figure 6(a)), indicating that suppression of HRG-*β*1-induced MMP-9 gelatinolytic activity by pterostilbene was due to the blockade of pro-MMP-9 protein production by HRG-*β*1.

In order to investigate whether the decreased HRG-*β*1-mediated MMP-9 protein level by pterostilbene was due to the down-regulation of MMP-9 mRNA, RT–PCR was performed on HRG-*β*1-stimulated MCF-7 cells in the presence or absence of pterostilbene. As shown in Figure 6(c), pterostilbene dose-dependently reduced HRG-*β*1-induced MMP-9 mRNA expression of MCF-7 cells, suggesting that pterostilbene might reduce the HRG-*β*1-induced MMP-9 expression through transcriptional regulation or modification of upstream kinase cascades.

### 3.7. Selective Inhibition of Pterostilbene on HRG-*β*1-Downstream Kinase Signaling Responsible for MMP-9 Induction

**Figure 7:**
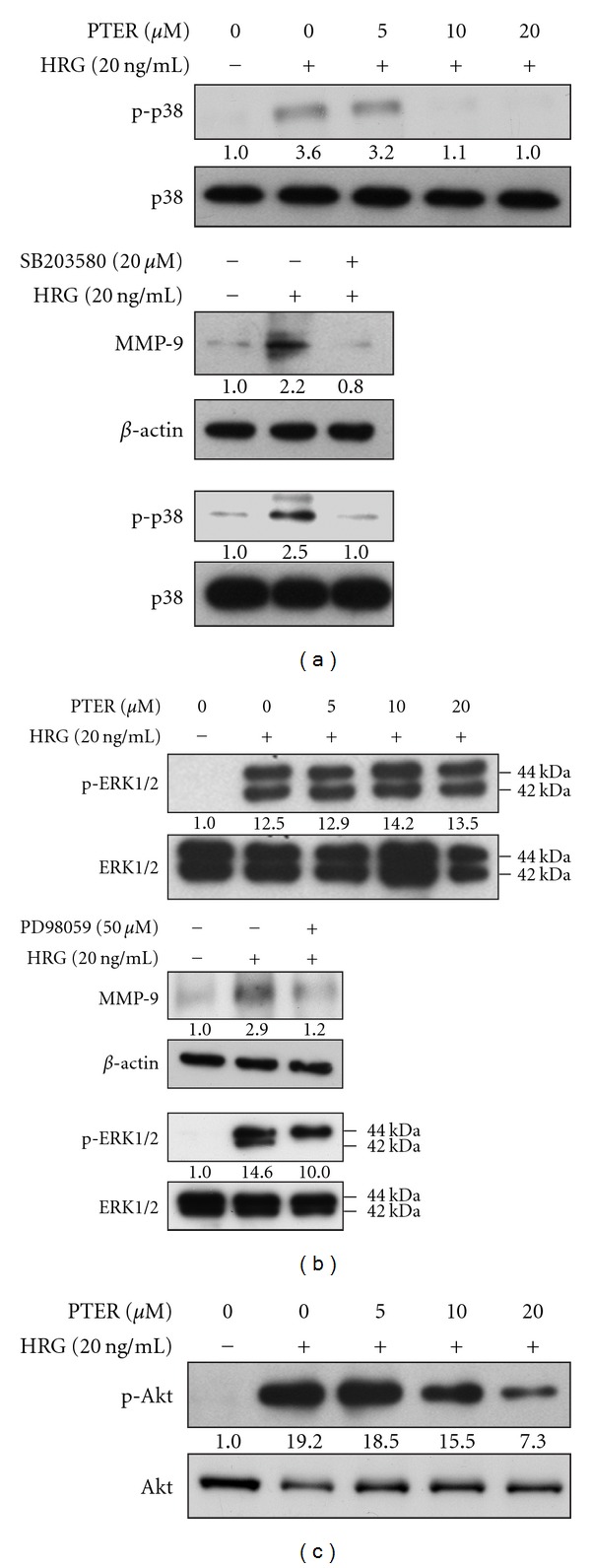
Effects of pterostilbene on the activation of p38 kinase, ERK1/2 and Akt in HRG-*β*1-stimulated cells. Serum-starved MCF-7 cells were pretreated with a variety of concentrations of pterostilbene, SB203580 (p38 kinase inhibitor, 20 *μ*M), or PD98059 (MEK inhibitor, 50 *μ*M) for 30 min and then stimulated by HRG-*β*1 (20 ng/ml) for 10 min. (a) Phosphorylated p38 kinase (p-38), (b) phosphorylated ERK1/2 (p-ERK1/2), (c) phosphorylated Akt (p-Akt) or MMP-9 was determined by western blotting. The native protein or *β*-actin was used as a loading control.

The activation of p38- and ERK1/2-MAP kinase cascade pathways are involved in the transcriptional up-regulation of MMP-9 by HRG-*β*1 [17]. To elucidate whether pterostilbene down-regulated HRG-*β*1-related MMP-9 expression by virtue of the modulation of activation of p38 kinase and ERK1/2, we performed immunoblotting to detect the effects of pterostilbene on HRG-*β*1-induced p38 kinase and ERK1/2 phosphorylation, which have direct positive correlation with their activation. SB203580 (p38 inhibitor) and PD98059 (MEK inhibitor) were used to identify the molecular signaling pathways by which HRG-*β*1 stimulates MMP-9 expression (Figures 7(a) and 7(b), resp.). Pterostilbene as effective as SB203580 (p38 inhibitor) reduced the phosphorylation of p38 kinase (Figure 7(a)); however, pterostilbene had no notable effect of on ERK1/2 phosphorylation (Figure 7(b)). These results suggested that pterostilbene blocked HRG-*β*1-mediated MMP-9 up-regulation through inhibiting p38 kinase rather than ERK1/2 in MCF-7 cells.

### 3.8. Dose-Related Repression on HRG-*β*1-Elicited Akt Phosphorylation/Activation by Pterostilbene

PI3K-Akt pathway is thought to be responsible for HRG-*β*1-associated cell proliferation. To evaluate whether the growth inhibition by pterostilbene may come from suppression of PI3K-Akt pathway, the effect of pterostilbene on HRG-*β*1-induced Akt phosphorylation (activation) was determined.  Figure 7(c) displays that pterostilbene caused a dose-dependent decrease in HRG-*β*1-induced Akt phosphorylation, correlated well with anti-proliferating effect (Figure 5). Combined with these findings, we suggest that the blockage of HRG-*β*1-induced proliferation by pterostilbene via an inhibitory modification of Akt may partially contribute to anti-metastatic and invasive capacity of pterostilbene in MCF-7 breast carcinoma cells.

## 4. Discussion

Clinically, up-regulation of MMPs is tightly associated with HER2 overexpression that imparts the aggressive and invasive potential to breast cancer with decreased survival [13]. Transfection of MCF-10A human breast epithelial cells with HER2 was sufficient to induce a more aggressive phenotype through MMP-9 and MMP-2 induction [14, 35]. These evidences suggest that HER2-bearing breast cancer cells, in part, acquire metastatic ability through up-regulation of MMPs. Thus, suppression of HER2-mediated MMP expression by chemopreventive agents may be an attractive strategy to reduce the incidence of malignant breast tumor development. Herein, the role of pterostilbene against HRG-*β*1/HER2-induced MMP-9 expression and invasion of MCF-7 human breast cancer cells has been first investigated. Our results showed that pterostilbene was able to suppress HRG-*β*1-mediated MMP-9 activation and expression with consequent suppression of cell invasion, migration and colony formation of MCF-7 cells (Figures 6, 2, 3, and 4, resp.).

Analysis of steady-state levels of MMP-9 mRNA indicated that pterostilbene inhibited HRG-*β*1-driven MMP-9 mRNA expression (Figure 6(c)). HRG-*β*1 is known for its ability to promote aggressive and invasive phenotypes of breast carcinoma by elevating the expression of MMP-9 via the MAPK and p38 kinase cascade pathways [17]. Both MAPK and p38 kinase pathways were reported to regulate the expression of MMP-9 by inducing AP-1-dependent promoter activation [36, 37]. In the light of these findings, HRG-*β*1-induced MMP-9 expression might be transcriptional. Thus, we postulated that the action mechanism of pterostilbene against cell invasion and metastasis of breast carcinoma might include the regulation or interference with these signaling pathways responsible for HRG-*β*1-mediated MMP-9 induction. Consistent with our assumption, pterostilbene has a dose-dependent inhibition on the phosphorylation of p38 kinase, a hallmark of kinase activation, but not the phosphorylation of ERK1/2 in HRG-*β*1-stimulated cells (Figures 7(a) and 7(b)), indicating that down-regulation of p38 kinase pathway might be one of the possible reasons for suppression of HRG-*β*1-elicted MMP-9 induction and invasion of breast cancer cells by pterostilbene.

Although anti-metastatic activity of pterostilbene on HRG-*β*1-stimulated cells can be explained to stem from its capacity of down-regulation of MMP-9 induction, dramatic inhibition of HRG-*β*1-induced colony formation and migration beyond the range of MMP-9 reduction was observed with treatment of pterostilbene at high concentrations in MCF-7 cells. It is likely that pterostilbene may affect other causal factors as well as MMP-9 expression that are responsible for colony formation and wound healing of MCF-7 cells triggered by HRG-*β*1. Interestingly, Tang et al. reported that resveratrol inhibits HRG-*β*1-mediated MMP-9 expression and cell invasion in MCF-7 cells [38], and it is known that resveratrol commits MCF-7 cells to apoptosis by accumulation oxidative stress and subsequent activation of different classes of MAP kinase pathways [39]. Moreover, our previous study found that pterostilbene was able to induce cell cycle arrest and apoptosis of human gastric carcinoma [40]. It raises the possibility whether pterostilbene acquires the ability to inhibit HRG-*β*1-associated cell invasion and cell transformation of breast carcinoma from its cytotoxicity. Our result indicated that HRG-*β*1 increased the cell proliferating rate in serum-starved MCF-7 cells and pterostilbene treatment reduced this elevated effect in a dose-related manner (Figure 5). Correlation with this growth inhibition, pterostilbene also dose-dependently blocked HRG-*β*1-induced phosphorylation (activation) of Akt, a prominent regulator for cell survival and transformation. On the basis of these findings, we infer that suppression of colony formation and wound healing by pterostilbene may partly result from growth inhibition in HRG-*β*1-stimulated cells, but the details of how these effects take place remain to be further elucidated.

**Figure 8:**
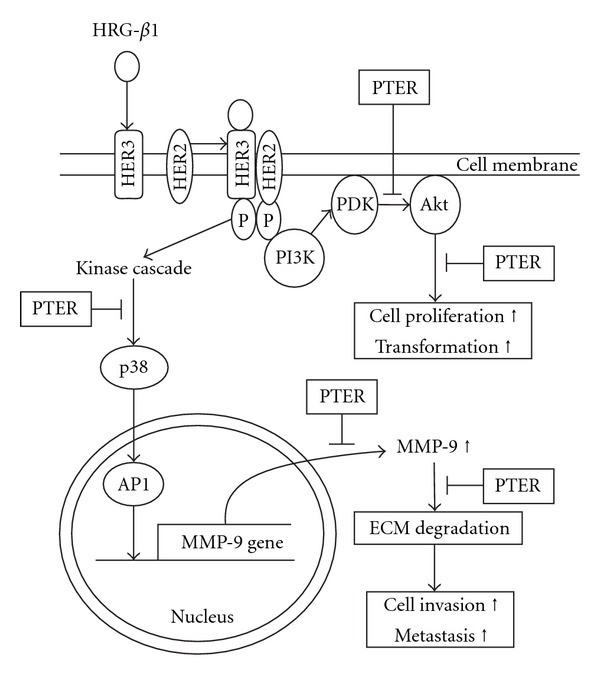
Schematic models depicting the effects of pterostilbene on HRG-*β*1-mediated aggressive phenotypes of MCF-7 cells. PTER: pterostilbene; HRG-*β*1: heregulin-*β*1; PI3K: phosphotidylinositol 3-kinase; PDK: phosphotidylinositol-3,4,5-triphosphate dependent kinase; p38 kinase, p38 MAP kinase; AP-1: activator protein-1; ECM: extracellular matrix. The arrow symbol represents direct or multistep activation; the T-like symbol represents site of inhibition by pterostilbene.

To sum up, the data given here provide clear evidence that pterostilbene abolishes the ability of HRG-*β*1-stimulated cells to metastasize and invade from two alternative pathways, as illustrated in Figure 8. One model of pterostilbene against metastasis is that pterostilbene exerts an inhibitory effect on p38 kinase pathway where it regulates HRG-*β*1-driven MMP-9 induction, invasion and migration of MCF-7 breast cancer cells. The other possible mechanism is that pterostilbene inhibits the PI3K/Akt pathway that is required for cell proliferation and transformation in response to HRG-*β*1. Under-standing the molecular basis of anti-tumor activity of pterostilbene, in conjunction with its low toxicity and non-mutagenic nature, will make it a potentially chemopreventive and therapeutic agent against some types of cancers.

## Funding

The National Science Council (NSC 95-2320-B-040-037; NSC 96-2320-B-040-023; and NSC 97-2320-B-040-015-MY3).
